# Annual prevalence of unmet healthcare need in Thailand: evidence from national household surveys between 2011 and 2019

**DOI:** 10.1186/s12939-021-01578-0

**Published:** 2021-11-12

**Authors:** Vuthiphan Vongmongkol, Shaheda Viriyathorn, Yaowaluk Wanwong, Waritta Wangbanjongkun, Viroj Tangcharoensathien

**Affiliations:** International Health Policy Program, Ministry of Public Health, Nonthaburi, Thailand

**Keywords:** Universal health coverage, Unmet need, Inequity, Access to health care, Thailand

## Abstract

**Background:**

Extending Universal Health Coverage (UHC) requires identifying and addressing unmet healthcare need and its causes to improve access to essential health services. Unmet need is a useful monitoring indicator to verify if low incidence of catastrophic health spending is not a result of foregone services due to unmet needs. This study assesses the trend, between 2011 and 2019, of prevalence and reasons of unmet healthcare need and identifies population groups who had unmet needs.

**Method:**

The unmet healthcare need module in the Health and Welfare Survey (HWS) 2011–2019 was used for analysis. HWS is a nationally representative household survey conducted by the National Statistical Office biennially. There are more than 60,000 respondents in each round of survey. The Organisation for Economic Co-operation and Development (OECD) standard questions on unmet need and reasons behind were applied for outpatient (OP), inpatient (IP) and dental services in the past 12 months. Data from samples were weighted to represent the Thai population. Univariate analysis was applied to assess unmet need across socioeconomic profiles.

**Results:**

The annual prevalence of unmet need between 2011 and 2019 was lower than 3%. The prevalence was 1.3–1.6% for outpatient services, 0.9% - 1.1% for dental services, and lower than 0.2% for inpatient care. A small increasing trend was observed on dental service unmet need, from 0.9% in 2011 to 1.1% in 2019. The poor, the elderly and people living in urban areas had higher unmet needs than their counterparts. Long waiting times was the main reason for unmet need, while cost of treatment was not an issue.

**Conclusion:**

The low level of unmet need at less than 3% was lower than OECD average (28%), and was the result of UHC since 2002. Regular monitoring using the national representative household survey to estimate annual prevalence and reasons for unmet need can guide policy to sustain and improve access by certain population groups.

## Background

Universal Health Coverage, one of the 2030 Sustainable Development Goals (SDGs), is a fundamental mechanism for improved health and well-being [1]. UHC means all people have access to essential health services without financial hardship [2] and is achieved through increasing population coverage, providing health services and ensuring financial protection [3]. UHC service coverage index and incidence of catastrophic health spending are two SDG 3.8 indicators [4].

Thailand achieved UHC in 2002 when the whole population was covered by one of the three public health insurance schemes: 7% of the total population by the Civil Servant Medical Benefit Scheme (CSMBS); 17%, who are private sector employees, are covered by Social Health Insurance (SHI); and the remaining 76% are covered by the Universal Coverage Scheme (UCS).

The members of each scheme are determined by their employment status. As access to health services become citizen’s entitlement; all citizens will be covered by one of the three schemes throughout their life. For example, the SHI members once unemployed are transferred to UCS; and vice versa, UCS members once employed in private sector are transferred to SHI. Dependents of CSMBS members who are beyond 20 years old once employed in private sector are transferred to SHI; or if unemployed, they are covered by UCS.

More than 90% of SHI members are the working age population (15–59 years), but this group accounts for around 59 and 54% of UCS and CSMBS respectively. While SHI has only 3% elderly members (> 60 years), the UCS and CSMBS have higher proportion of older people, 19 and 36% respectively [5]. In addition, about two-thirds of the UCS members live in the rural area, but two-thirds of the SHI and the CSMBS members resided in urban areas [6]. There are also socio-economic differences. Analysis from 2019 Health and Welfare Survey shows that more than 50% of the CSMBS members belong to the fifth (richest) wealth quintiles; 27.6% of SHI and 14.9% of UCS members are in richest quintiles. In contrast, 21.7, 19.5 and 4% of UCS, SHI and CSMBS members belong to the poorest wealth quintile [6].

All three schemes offer comprehensive benefits package including outpatient, inpatient and all medicines listed in the national essential medicines [7, 8], and all high-cost but effective interventions. The comprehensive benefit package by all three scheme and no copayment at point of service result in low incidence of catastrophic health spending, as measured by out-of-pocket payments exceeding 10% of household total consumption expenditure. It reduced from 6.0% in 1996 to 2.2% in 2017 [9, 10].

Unmet healthcare need, defined as people who need health services but do not use them for whatever reasons [11], is a key indicator for monitoring access to health services as it reflects the gaps of access to care such as availability, geographical accessibility, financial accessibility, poor quality of care that people do not trust. Countries having low incidence of catastrophic health spending can be a result of unmet need due to inability to use health services which results in disability or mortality. Also, unmet need can be applied to monitor access to care by vulnerable populations or specific diseases [8]. In this regard, WHO member states who committed to UHC should also consider monitoring the unmet need as a flip side of catastrophic health expenditure in SDG target 3.8.2 [12]. Literature shows that high-income OECD countries conduct regular survey and produce report on annual prevalence of unmet need [13]. Thailand is one of a few low- and middle-income countries (LMICs) that capture unmet need through a biennial national household survey. The first monitoring unmet need as part of the Panel Socio-Economic Survey in 2010 conducted by the National Statistical Office (NSO) was published in 2012 [8]. Learning from OECD experiences, an unmet module was embedded into the 2011 HWS.

NSO initiated HWS in 1974 for monitoring health risks and service use every 5 years until 2001. In responses to requests by the International Health Policy Program of the Ministry of Public Health to monitor the impact of UHC launched in 2002 on households; NSO decided to conduct HWS annually between 2003 and 2007 and biennially from 2009 onward. Not until 2011 that an unmet need module was embedded into the HWS with the aim to monitor the annual prevalence of unmet need. In 2017, unmet need using the 2015 HWS was published in Thai journal [14]. Apart from the two publications of unmet need in 2010 and 2015, there was no continued update of unmet need in Thailand.

Given the achievement of full population coverage since 2002 and the comprehensive benefits package without copayment at point of services by all schemes; evidences showed that health care utilization was notably different across geographical regions with a low OP utilization and IP admission rate in the central region and Bangkok--the capital of Thailand [6]. Hence, a few policy questions are raised; 1) What is the prevalence of unmet needs in the population? 2) How unmet need differs across the regions? 3) What are the causes of these unmet needs? and 4) Who are affected by unmet need?

To respond to these policy questions, this study aims to assess the prevalence and trend of unmet need between 2011 and 2019, reasons of unmet need, and identifies population groups who are affected. In this study, unmet healthcare need includes outpatient, inpatient and dental services. Findings can be useful for LMICs to initiate regular monitoring which improve access to health services in their quest for UHC.

## Method

### Data source

This study used data from the HWS. The sample size of individuals in 2011, 2013, 2015, 2017 and 2019 were 71,847, 71,533, 139,858, 65,781 and 63,594 respectively.

### Data collection

All respondents were asked about their unmet need of outpatient, inpatient and dental health services in the last 12 months. As proxy respondent, the heads of household were allowed to provide information on behalf of children under 15 years old, elderly, and members who were absent on the interview date. This standard question is used: “During the last 12 months, was there any time when you personally, really needed a medical examination or treatment for a health problem (as an outpatient, inpatient or dental health services), but you did not receive it? Yes/No”.

The respondents who reported unmet need in the last 12 months were asked about their reasons- “What was the main reason for not receiving treatment/services at that time”. There were 9 different reasons which were: “Could not afford the cost of treatment”/ “Could not afford travel cost” / “Long waiting time”/ “Difficulty travelling or living far away from facilities”/ “No time to go get treatment”/ “Do not trust or feel confident with facilities or providers”/ “Did not know where to go receive treatment”/ “No accompany person to health facilities”/“Other reasons” for each of the three conditions (outpatient, inpatient and dental health services). One additional reason “Provider cannot provide proper service” was given in relation to inpatient and dental services.

During the fieldwork in 2017 and 2019, there was a technical error in collecting the unmet need for inpatient and dental services. Data from the field were collected by using electronic tablets. The error happened when data were downloaded from tablets onto the national database system at NSO; outpatient data were downloaded as inpatient and dental services throughout. To fill the missing unmet need of inpatient and dental care in 2017 and 2019, the trend projection applied the best model between linear and exponential regression for 2017 and 2019 based on 2011, 2013 and 2015 base years. However, reasons for unmet need cannot be projected; hence they are missing from the 2017 and 2019 surveys.

### Analytical methods

The annual prevalence of unmet need was estimated using sampling weight to represent the entire Thai population. NSO provided these weights according to the probability of each individual being randomly selected. A Principal Component Analysis (PCA) was applied to compute an asset index score of each household based on ownership of durable and housing characteristics. The wealth quintile was categorized into five groups based on their asset index score of each household ranked from the lowest to the highest. The first quintile was the poorest (20% of the lowest asset index) and the fifth quintile was the richest (20% of the highest asset index). All variables were presented as percentage and univariate analysis with chi-square statistic applied to test the statistically significant difference.

## Results

### Characteristics of the study population

The sample size varied between 63,594 and 139,858 adults who responded to the face-to-face interview surveys. These samples were weighted to represent the whole population. Table 1 illustrates demographic and other profiles such as insurance coverage and chronic conditions. Similar profiles were observed throughout five waves of survey.

**Table 1:** Sample demographic and other profiles

Population characteristics	Year				
	2011	2013	2015	2017	2019
Sample size	71,847	71,533	139,858	65,781	63,594
Number population (N)	67,495,323	66,263,166	67,163,733	67,572,274	67,921,857
Sex (%)					
Male	49.1	48.9	48.8	48.8	48.7
Female	50.9	51.1	51.2	51.2	51.3
Age group (%)					
0–5 year	7.5	6.9	6.8	6.7	6.5
6–14 year	12.7	11.6	11.1	10.7	10.3
15–44 year	44.2	45.9	44.7	43.7	42.8
45–59 year	23.5	21.4	22.1	22.5	22.7
60 year and over	12.1	14.2	15.4	16.5	17.8
Municipal area (%)					
Urban	34.3	44.5	44.5	44.7	44.8
Rural	65.7	55.5	55.5	55.3	55.2
Region (%)					
Bangkok	10.2	12.8	12.8	12.9	13.0
Central	23.8	28.3	28.6	29.0	29.4
North	18.1	17.5	17.1	16.9	16.7
Northeast	34.0	27.9	27.9	27.6	27.3
South	14.0	13.6	13.6	13.7	13.8
Health Insurance (%)					
UCS	80.1	76.1	75.8	75.0	75.1
SHI	11.3	15.3	16.1	17.0	17.6
CSMBS	8.6	8.6	8.1	8.0	7.3
Education (%)					
No education	4.3	4.9	4.4	4.6	4.3
Primary school and below	57.5	52.6	50.6	49.9	48.2
Secondary school and diploma	29.5	31.5	33.5	34.1	35.1
Bachelor’s degree and above	8.6	10.9	1.0	11.1	12.1
Others and unknown	0.1	0.2	0.5	0.4	0.3
Marital Status (%)					
Single	26.0	28.4	28.3	29.0	29.8
Married	63.1	60.4	60.3	59.0	57.8
Widowed	7.2	7.4	7.6	7.8	7.9
Divorce / separated	3.8	3.8	3.8	4.2	4.5
Having chronic diseases (%)					
No	84.0	82.6	82.5	81.4	82.2
Yes	16.0	17.4	17.5	18.6	17.8

### Unmet need

#### Total unmet need and unmet need of outpatient, inpatient and dental services

**Fig. 1:**
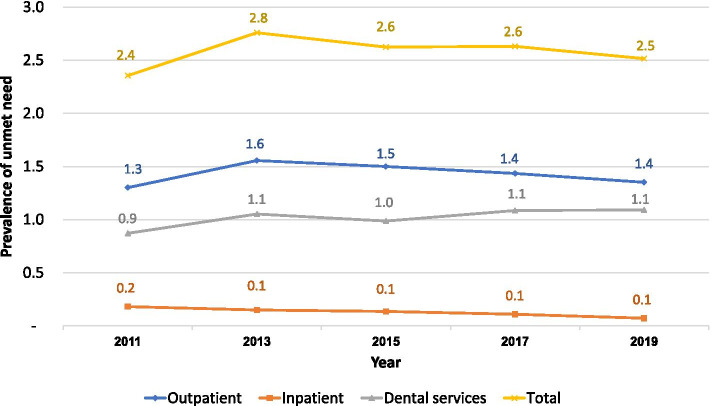
Annual prevalence of unmet need by three types of services, 2011-2019

The annual prevalence of total unmet need, including OP, IP and dental services slightly increased from 2.4% in 2011 to 2.8% in 2013 and decreased to 2.5% in 2019. In 2019, the unmet need for OP (1.4%) was higher than dental services (1.1%); while IP has the least prevalence 0.1%. See Fig. 1.

#### Total unmet need: which population groups are affected

Univariate analysis shows significant increased total unmet need with age. The total unmet need in 2019 was high among elderly (4.8%) and comparatively low in children under five (0.7%). People living in the Southern region had twice higher total unmet need (4.3%) in 2019 than other regions. The members of CSMBS had the lowest total unmet need compared with the other two insurance schemes. The richer wealth quintiles had lower total unmet need than the poorer quintiles. The poorest quintile had twice the total unmet need than that of the richest quintile in 2019; and people who did not have chronic disease had lower total unmet need than those with these conditions; see Table 2.

**Table 2:** Annual prevalence of total unmet need by socio-economic profiles, 2011–2019

Sub-group variables		Total unmet need							
		2011		2013		2015		2017^a^	2019^a^
		%	P-value	%	P-value	%	P-value	%	%
**Overall unmet need**		**2.4**		**2.8**		**2.6**		**2.6**	**2.5**
Sex	Male	2.0	0.013*	2.4	0.348	2.4	0.424	2.6	2.8
	Female	2.3		2.6		2.4		2.6	2.6
Age group	0–5 year	1.1	0.000***	1.0	0.000***	0.9	0.000***	0.8	0.7
	6–14 year	1.1		1.1		1.2		1.2	1.2
	15–44 year	1.8		2.2		2.0		2.3	2.4
	45–59 year	3.2		3.3		3.2		3.2	3.1
	60 year and over	3.3		4.3		4.0		4.5	4.8
Areas of residence	Urban	2.3	0.002**	2.6	0.002**	2.5	0.004**	2.6	2.7
	Rural	2.1		2.5		2.4		2.6	2.7
Geographical region	Bangkok	3.4	0.000***	3.3	0.000***	3.1	0.000***	2.9	2.7
	Central	2.3		2.1		2.4		2.4	2.5
	North	2.7		2.4		2.5		2.3	2.2
	Northeast	1.7		1.8		2.0		2.1	2.3
	South	1.6		4.2		2.6		3.8	4.3
Health Insurance	UCS	2.2	0.002**	2.6	0.002**	2.5	0.000***	2.7	2.9
	SHI	2.2		2.6		2.2		2.4	2.4
	CSMBS	2.0		1.9		1.8		1.7	1.7
Asset index quintile	Quintile 1	3.0	0.000***	3.4	0.000***	3.3	0.000***	3.6	3.8
	Quintile 2	2.1		2.7		2.4		2.8	2.9
	Quintile 3	2.1		2.4		2.3		2.4	2.4
	Quintile 4	1.7		2.2		2.1		2.4	2.6
	Quintile 5	2.0		1.9		1.9		1.8	1.8
Chronic diseases	No	1.8	0.000***	1.9	0.000***	1.9	0.000***	0.0	0.0
	Yes	4.4		5.4		5.0		5.6	5.9

Note: 1) ^a^The statistic test was not applied for projected unmet needs in 2017 and 2019. 2) * *P* ≤ 0.05 ** *P* ≤ 0.01 *** *P* ≤ 0.001

#### Specific unmet need: which population groups are affected

We conducted univariate analysis to assess the association between unmet need of OP, IP and dental services and other socio-economic parameters. The prevalence of unmet needs between male and female for the three services was not statistically significantly different; although females have lower unmet need than males. There were statistically significant differences for other independent variables (*p*-value < 0.05) (Table 3).

**Table 3:** Annual prevalence of unmet needs for outpatient, inpatient and dental services, 2019

Sub-group variables		Outpatient care		Inpatient care	Dental care
		%	P-value	%	%
**Overall unmet need**		**1.4**		**0.1**	**1.1**
Sex	Male	1.5	0.626	0.1	1.2
	Female	1.2		0.1	1.1
Age group	0–5 year	0.1	0.000***	0.1	0.6
	6–14 year	0.5		0.1	0.6
	15–44 year	1.3		0.0	1.0
	45–59 year	1.8		0.1	1.5
	60 year and over	1.9		0.6	1.5
Areas of residence	Urban	1.5	0.002**	0.0	1.2
	Rural	1.2		0.2	1.1
Geographical region	Bangkok	1.9	0.000***	0.0	0.7
	Central	1.6		0.2	1.5
	North	1.3		0.2	1.2
	Northeast	0.8		0.0	0.5
	South	1.6		0.3	1.8
Health Insurance	UCS	1.4	0.000***	0.1	1.2
	SHI	1.5		0.0	1.0
	CSMBS	0.8		0.2	0.7
Asset index quintile	Quintile 1	2.1	0.000***	0.2	1.9
	Quintile 2	1.3		0.2	1.2
	Quintile 3	1.0		0.1	0.9
	Quintile 4	1.4		0.0	1.2
	Quintile 5	0.9		0.1	0.6
Chronic diseases	No	1.0	0.000***	0.0	1.0
	Yes	2.8		0.6	1.9

Note: The statistic test was not applied for projected data on unmet needs of inpatient and dental services in 2019. ** *P* ≤ 0.01, *** *P* ≤ 0.001

The level of unmet needs increase with age; unmet need among older persons was more than double that of the youngest group, notably for OP and IP services. The urban household had higher unmet needs for OP services, but lower IP services compared with the rural population. The highest unmet needs for OP care were in the Greater Bangkok area; but this area had relatively low unmet need for inpatient and dental care; while unmet need for hospitalization and dental services were highest in the South.

Clearly, wealth influenced unmet need. The poorest quintile had twice higher unmet need for OP and IP care, and three times higher for dental services than the richest quintile. Concerning the disparity across three health insurance schemes, access to OP and dental services by CSMBS members was better than others; the reported unmet need for OP services by the UCS was relatively high; despite a generally very low level of unmet need across three schemes. People living with chronic diseases who had greater health needs reported a higher prevalence of unmet need compared with individuals with no chronic illnesses; this was across all services.

#### Reasons for unmet need

The top three reasons for unmet outpatient need between 2011 and 2019 were long waiting time, no time to seek care by the population and inconvenient transportation. In 2019, these three reasons accounted for 71.9% of total causes. The unmet need for outpatient services caused by unaffordable treatment cost was low, 1.1% in 2019, See Table 4.

**Table 4:** Reasons for unmet need for outpatient services (%), 2011–2019

Reasons of unmet outpatient needs	2011	2013	2015	2017	2019
long waiting time	na	22.7	32.4	29.5	38.4
No time to seek care	30.1	24.0	23.6	27.8	19.8
Inconvenient transportation	14.7	12.3	10.7	10.0	13.7
No companions to seek care	9.9	6.0	6.3	4.6	8.1
Do not trust in providers	6.3	3.6	3.8	2.9	3.2
Cannot afford transportation cost	0.8	1.9	2.6	3.1	1.7
Cannot afford treatment cost	1.1	2.7	2.5	2.2	1.1
Do not know which provider is available	2.1	0.9	0.9	0.6	0.6
Do not believe that treatment can solve health problems	7.3	na	na	na	na
Other reasons	27.8	25.9	17.2	19.3	13.3

The top three reasons for unmet needs of inpatient services between 2011 and 2015 were inconvenient transportation, no time to seek care and long waiting time; accounting for 43.9% of total causes. Note that 7.5% in 2015 of those reported inpatient unmet needs said they cannot afford to pay treatment cost despite the fact of comprehensive benefits package and literally no co-payment. See Table 5.

**Table 5:** Reasons for unmet need for inpatient services (%), 2011–2015

Reasons of unmet inpatient needs	2011	2013	2015
Inconvenient transportation	11.0	17.3	16.0
No time to seek care	16.4	29.6	14.9
long waiting time	na	6.2	13.1
Do not trust in providers	22.7	8.0	10.6
No companions to seek care	3.0	8.1	9.5
cannot afford treatment cost	6.1	7.3	7.5
beds not available	na	9.3	4.9
cannot afford transportation cost	0.7	2.3	4.4
Do not know which provider is available	5.9	–	0.6
No dental service provided in living area	5.0	na	na
Do not believe that treatment can solve health problems	10.9	na	na
Other reasons	18.3	11.7	18.6

Note: data is missing for 2017 and 2019

The top three reasons for unmet needs of dental services between 2011 and 2015 were no time to seek care, long waiting time and inconvenient transportation; altogether 69.5% of total causes. Unmet need caused by cost of treatment was 4.8% of total reasons. See Table 6.

**Table 6:** Reasons for unmet need for dental services (%), 2011–2015

Reasons of unmet dental needs	2011	2013	2015
No time to seek care	43.8	31.2	33.5
long waiting time	–	21.2	28.4
Inconvenient transportation	10.1	5.1	7.6
cannot afford treatment cost	na	8.5	4.8
No companions to seek care	5.1	4.2	3.5
Do not trust in the providers	7.2	3.5	2.4
No dental service provided in the area	3.5	1.4	2.2
cannot afford transportation cost	1.2	1.3	1.3
Do not know which provider is available	2.4	2.4	0.4
Do not believe that treatment can solve health problems	2.5	na	na
Other reasons	24.2	21.2	16.0

Note: Data missing in 2017 and 2019

Comparing unmet needs across three services caused by cost of treatment, OP services had the lowest 1.1% of all causes; though it is higher for inpatient (7.5%) and dental services (4.8%).

## Discussions

Prevalence of unmet need for health services by socio-economic stratification is an important indicator to monitor inequity in access to health care. This study analyses the trend of unmet need for OP, IP and dental care services between 2011 and 2019 when the UHC system became mature.

The low prevalence of total unmet need for these services is a result of extensive coverage of health service, notably primary health centres in all sub-districts and district hospitals in all districts nationwide. Annual prevalence of unmet healthcare need, 2.5% in 2019, was lower than the 28% average in OECD countries ranging from 10% in Norway to 47% in Ireland [15]. A major cause of unmet need in OECD countries was long waiting times [15]; which is similar to this study. However, the financial distress--a major reason of unmet need in OECD countries [15] was still a reason for unmet need of inpatient and dental services; though the prevalence was much lower than OECD average. United Kingdom, having long history of UHC implementation [16], had low level of unmet need of medical and dental services caused by financial barrier [15]. This information implies the contribution of comprehensive benefits package and availability of health services to low prevalence of unmet needs.

Three decades prior to UHC in 2002, Thailand invested on large-scale health infrastructure and human resources for health to establish the close-to-client district health system [17]. This ensured that services are available and accessible close to people’s home with little time and transportation cost. The full geographical coverage of district hospitals, in all districts, was achieved in 1990 [17]. Over the next 10 years, full geographical coverage with health centres as the first point of contact in each sub-district has been achieved [17]. The development of health infrastructure was supported by the scaling up of health workforce training, deployment and distribution to create functioning district health systems [17].

Comprehensive benefits packages are covered by all three health insurance schemes with no copayment at the point of service [7]. This is an important factor to improve access to health services and strengthen financial protection. The data shows that out-of-pocket payments significantly decreased from 34% of current health spending (CHE) in 2000, before the UHC era, to 11% of CHE in 2018 [18].

Thailand achieved high level of health development as reflected by near 100% population coverage by three insurance schemes [17], low incidence of catastrophic health spending [9], better health outcomes [19] such as life expectancy increased from 71 years in 2001 to 77 years in 2019, decreased under-five mortality from 22 per 1000 live births in 2000 to 9 per 1000 live births in 2019, decreased maternal mortality ratio from 43 per 100,000 live births in 2001 to 37 per 100,000 live births in 2017.

Despite low level of overall unmet need, the trend between 2011 and 2019 had not significantly improved while inequitable gaps across different population groups and socio-economic status remained. Also, we find higher unmet need among people living in Bangkok, the elderly and members of UCS. This evidence contributes to policy design to minimize the gaps. Long waiting times for outpatient services became more prominent in 2019, especially in urban setting such as Bangkok with inadequate primary healthcare services. Waiting time is the result of an imbalance between demand and supply of primary care. Notably, the population density of Bangkok is 28 times higher than the national average [20], and primary health care is fragmented and provided by different uncoordinated providers such as private clinics and outpatient departments of tertiary care in public and private hospitals [21]. The network of primary care is not organized by the Ministry of Public Health as it is in other provinces with a strong foundation of primary care provided by district health systems [21]. Unmet need due to long waiting times in Bangkok led to a major investigation in 2020 in order to reform the provision and financing of primary care through contractual agreements with private clinics and hospitals in order to minimize the unmet needs [22]. Also, the dispensing and refill medication by private pharmacies for UCS patients in Bangkok and other regions was launched in 2020 [23].

The main reasons for high prevalence of unmet need in all three services among elderly people were waiting times and lack of transport. This is a key policy concern in a rapid ageing society with almost 20% of the total population being elderly in 2022. Thailand is projected to become a super-aged society (where more than 20% of population is 65 years and older) by 2033 [24]. High prevalence of diabetes and hypertension was reported among Thai elderly people during 2002–2017 [25], and delayed access to OP services may result in hospitalizations and higher co-morbidity and mortality. Home- and community-based health care is gradually being established to respond to the health needs of older persons.

The inequitable distribution of unmet need across income groups confirms previous studies [8, 26]. The higher unmet need for all services among the poorest quintile confirms findings from OECD countries [13, 15]. Disparity of unmet need across three insurance schemes is the result of disparity of socio-economic status. UCS members have higher proportion of the poorest quintile. While 53.6% of CSMBS members belong to the richest quintile, only 14.9% of UCS members are in the richest quintile [6].

The UCS and SHI members had higher unmet need for dental services than the CSMBS mainly due to long queues and no time to seek care. Although a comprehensive benefits package is provided by three health insurance schemes, some different provisions and payment methods affect the use of dental services. The SHI members can use the services only through contractor providers at the public or private hospitals they are registered with. CSMBS has a free choice to use any public provider as no registration to a provider is required. UCS members need to seek care at the registered contractor provider network, which are mostly district health systems at the provincial level, or contractor private clinics, or public hospitals in Bangkok [27].

However, SHI covers only fillings, tooth extraction, scaling, and impact wisdom teeth extraction with a maximum annual reimbursement of 900 Bath (around 30 US$) [27]; the balance beyond the reimbursement level is responsible by SHI members. The UCS applies capitation with registration to a contractor provider network, while the CSMBS uses fee-for-service for both OP and dental services [7]. These variations could lead to inequity of access to dental services across three schemes. Harmonizing the benefits package and payment methods across insurance schemes may minimize unmet need, although demand-side factors such as education and socio-economic status are key factors of unmet need [8, 27].

This study suggests that regular monitoring of unmet need can inform policy targeting the improvement of equitable access to different types of health services (such as outpatient, inpatient and dental services) as it identifies who are affected and the reasons behind. Unmet need is important evidence to confirm if the low incidence of catastrophic health expenditure is “real” and not because of unmet need where people do not use health service, no out-of-pocket payment and hence no catastrophic health spending.

There are some limitations of this study. First, though the strength of this survey is its national representativeness and minimum sampling bias, a non-sampling bias from a 12-month recall period is a limitation of under-reporting biases. However, 12-month recall period is applied by OECD. Second, projecting unmet need for IP and dental services in 2017 and 2019 using 2011, 2015 and 2017 trends may not reflect the real unmet need. Further there is no data on reasons of unmet need for IP and dental services in 2017 and 2019. Third, comparison of unmet needs and after the introduction of UHC in 2002 is not possible as the unmet need questions had been embedded in HWS in 2011. Lastly, the reason for unmet need was selected from the primary reason provided by households.

## Conclusion and recommendations

 Though the overall unmet need was low as a result of adequate access to well-functioning district health systems and easy access by citizens at provincial level, but lack of primary care in Bangkok leads to higher unmet need for people in Bangkok. Unmet need from cost is not a problem due to a comprehensive benefits package offered by three insurance schemes. Despite these achievements, we found that long waiting times was the main reason for unmet need. Improve service provision through better appointment systems will minimize the queue.

The good collaboration between the Ministry of Public Health through International Health Policy Program and National Statistic Office contributes to embedding the unmet need module into the HWS. It is an intelligence system to monitor and check that the low incidence of catastrophic spending is not a result of foregone services due to unmet needs.

The unmet healthcare need assessment from Thailand can be a good lesson for other LMICs in their paths towards UHC to prove the low prevalence of catastrophic health spending is not from inability to use service; and embedding an unmet need module in their national household surveys is a key platform for routine monitoring.

From this study we recommend the following.

First, continue monitoring unmet need and introduce policy to minimize the inequity of unmet needs across population groups and socio-economic status. As the prevalence was very low, 2.5% in 2019; it is unlikely to further reduce.

Second, strengthen primary healthcare by prioritizing Bangkok metropolitan area through establishing new primary healthcare or contracting private clinics. Bring health services to elderly through home visits in particular the home-bound and bed-bound chronic ill patients.

Third, initiate and evaluate policies to reduce waiting time. For example, the use of open access scheduling for primary care appointments, capitalize the extensive coverage of mobile phone and emails for follow-up consultation [28].

Fourth, assess the relationship between geo-spatial distribution of healthcare facility and unmet needs, which support specific location for investment in primary health care.

Finally, continue embedding unmet need module in the biennial HWS is a sustained platform for regular monitoring and timely policy intervention. LMIC may apply this strategy to improve performance of health service provision along the pathway towards UHC.

## Data Availability

All data used in this study are available from NSO with some restrictions. The data are not publicly available; using the data for public non-profit purpose is approved by the NSO.
